# Mitochondrial dynamics in the adult cardiomyocytes: which roles for a highly specialized cell?

**DOI:** 10.3389/fphys.2013.00102

**Published:** 2013-05-10

**Authors:** Jerome Piquereau, Fanny Caffin, Marta Novotova, Christophe Lemaire, Vladimir Veksler, Anne Garnier, Renee Ventura-Clapier, Frederic Joubert

**Affiliations:** ^1^Department of Signaling and Cardiac Pathophysiology, U-769, INSERMChâtenay-Malabry, France; ^2^IFR141, Université Paris-Sud Châtenay-Malabry, France; ^3^Department of Cellular Morphology, Institute of Molecular Physiology and Genetics, Slovak Academy of SciencesBratislava, Slovak Republic

**Keywords:** mitochondrial dynamics, cardiomyocytes, adult, energetic metabolism, cytoarchitecture

## Abstract

Mitochondrial dynamics is a recent topic of research in the field of cardiac physiology. The study of mechanisms involved in the morphological changes and in the mobility of mitochondria is legitimate since the adult cardiomyocytes possess numerous mitochondria which occupy at least 30% of cell volume. However, architectural constraints exist in the cardiomyocyte that limit mitochondrial movements and communication between adjacent mitochondria. Still, the proteins involved in mitochondrial fusion and fission are highly expressed in these cells and could be involved in different processes important for the cardiac function. For example, they are required for mitochondrial biogenesis to synthesize new mitochondria and for the quality-control of the organelles. They are also involved in inner membrane organization and may play a role in apoptosis. More generally, change in mitochondrial morphology can have consequences in the functioning of the respiratory chain, in the regulation of the mitochondrial permeability transition pore (MPTP), and in the interactions with other organelles. Furthermore, the proteins involved in fusion and fission of mitochondria are altered in cardiac pathologies such as ischemia/reperfusion or heart failure (HF), and appear to be valuable targets for pharmacological therapies. Thus, mitochondrial dynamics deserves particular attention in cardiac research. The present review draws up a report of our knowledge on these phenomena.

## Introduction

Mitochondria, which have long been regarded only as energy producers, are actually recognized at the crossroads of many cellular functions. Obviously, they play a crucial role in energy production in cells, but they are involved in other phenomena such as ion homeostasis, free radical production, and ultimately cell death. Many of their characteristics, as morphology, location in the cell, proximity to other organelles are important parameters that have to be considered to understand the mitochondrial functions. This is particularly true in the adult cardiac cells where mitochondria, which produce 90% of ATP, occupy 30% of cardiac cell volume and are embedded in a dense and complex organization. This interlinking reflects the challenging function of the heart which requires rhythmic contractions of the pump throughout the life, and consequently needs a fast and effective intracellular energy delivery to the ATP consumers of the cardiomyocyte (for review, see Ventura-Clapier et al., 2011). Moreover, the main phosphorylated metabolites do not vary with the increase in work (Balaban, 2012). This metabolic homeostasis and the tight coupling between mitochondria and ATP consumer sites are two of the peculiarities of the cardiac cell and require an optimized cellular organization to ensure efficient energy fluxes. Any modification of the cellular architecture, but also of the internal organization of mitochondria could thus impair cell energetic and as a consequence cell function.

In the majority of cells, mitochondria are able to adjust their morphology and their location depending on energy needs and metabolic conditions (Hackenbrock, 1966; Bereiter-Hahn, 1990; Karbowski and Youle, 2003; Rossignol et al., 2004; Mannella, 2006; Benard et al., 2007; Soubannier and McBride, 2009). This “mitochondrial dynamics” seems to be particularly important during cell division and for mitochondrial quality control; it may also play a role under pathological conditions. At some point, mitochondrial network morphology actually is the result of several processes, including fusion and fragmentation of the organelles which are usually controlled by a complex protein machinery (for review, see Liesa et al., 2009). It is generally admitted that a connected mitochondrial network is observed in active metabolic cells (Skulachev, 2001) while the mitochondria are rather fragmented in quiescent cells (Collins et al., 2002). However, it should be kept in mind that mitochondria exhibit a high structural and functional tissue specificity in connection with cell functions. Thus, any finding on mitochondrial organization and functioning cannot be directly extrapolated before being considered in the framework of the considered cell or tissue. For example in adult cardiac cells, the relationship between mitochondrial morphology and function does not seem to be rigorous, since cardiomyocytes are metabolically active but exhibit an apparently fragmented network (Kuznetsov et al., 2009).

Mitochondrial dynamics, however, depends on the cellular environment and architecture constraints. In adult cardiomyocytes, the large amount of myofilaments, the presence of a rigid cytoskeleton and the densely packed mitochondrial network clearly impedes mitochondrial movements (Vendelin et al., 2005). Moreover, the arrangement of the different organelles between them is so crucial for cardiac cell function that mitochondrial morphology has to be efficiently controlled (Wilding et al., 2006; Piquereau et al., 2010). Although abundance of proteins of the mitochondrial dynamics can appear paradoxical in cardiac cells, where the mitochondrial network appears to be frozen, it may be less rigid than believed. Proteins of the mitochondrial dynamics are involved in multiple processes and can thus be important for cardiac physiology out of their role in mitochondrial network organization. In this review, we present some evidences for the importance of these proteins in physiological and pathological situations.

## Mitochondrial functions in the adult cardiac cell: tangled in a complex architecture

### Role of the internal organization of mitochondria

Since the discovery of the chemiosmotic mechanism of ATP synthesis by PD Mitchell in the 1960s, the importance of mitochondrial internal organization and of a local regulation of energy production has been pointed out by many authors. Indeed, organization of internal membranes of mitochondria is critical for an optimal function of respiratory complexes and ATP formation (Davies et al., 2011). The existence of a large inner membrane folded in cristae, where oxidative-phosphorylation coupling occurs, is a prerequisite for local proton gradient generation and maximal ATP synthase functioning (Strauss et al., 2008). Phospholipids, and in particular cardiolipins, are involved in the formation of these cristae (Khalifat et al., 2008). However, the all set of elements inducing cristae generation is far from being elucidated. For example, the presence of oligomers of ATP synthase (Davies et al., 2012) and of dynamin proteins (Hinshaw, 2000) could also play a role in the cristae organization. Different dynamin proteins, such as the optic atrophy protein 1 (Opa1) or Mitofilin, have been recognized as important actors regulating cristae formation (Frezza et al., 2006; Hoppins et al., 2011a). Those cristae are dynamic structures which can rapidly and reversibly fuse and divide, depending on energetic state (Mannella, 2006), from orthodox to condensed conformations upon activation of ATP synthesis (Hackenbrock, 1966; Mannella, 2008). Thus, optimal mitochondrial function seems to require the morphological control of inner membrane organization. This is particularly evident for mitochondria of the adult cardiomyocyte that exhibit the highest density of cristae (Vafai and Mootha, 2012). Finally, it is also well-known that changes in mitochondrial morphology can play a role in different events, in particular in mitochondrial permeability transition pore (MPTP) function, and in apoptosis (Nogueira et al., 2005; Wasilewski and Scorrano, 2009; Campello and Scorrano, 2010). So it is now evident that all the phenomena that modify mitochondrial morphology can modify mitochondrial functions, and possibly cell functions.

Another important feature of mitochondria, especially in cardiac cells, is the existence of local control of energy production in the intermembrane space by different phosphotransfer enzyme systems. For example, we and others have shown that the mitochondrial creatine kinase isoform (miCK), which is located in the vicinity of the adenine nucleotide translocase (ANT), allows a local supply of ADP, a local control of proton concentration, and an efficient transfer of energy via phosphocreatine (PCr) diffusion (Joubert et al., 2000, 2001a, 2002a; Saks et al., 2000; Guzun et al., 2012). Such enzymes also play a role in the structural organization of the intermembrane space by bridging inner and outer membranes (Wallimann et al., 1992; Speer et al., 2005). Any modification of this energetic microdomain organization by alteration of mitochondrial morphology could affect local regulation of energy production.

### Importance of cell architecture for energy transfer and communication between organelles

**Figure 1:**
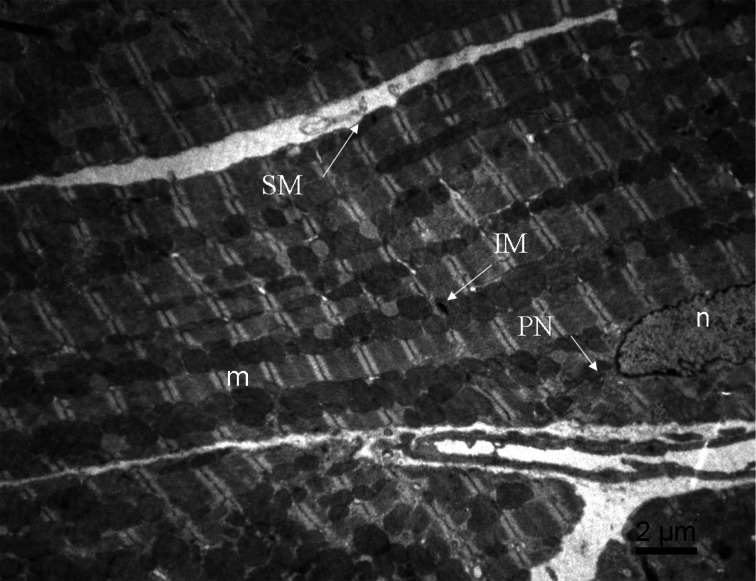
**Image of adult mouse cardiomyocyte obtained by electron microscopy.** Three subpopulations of mitochondria are observed: intermyofibrillar mitochondria (IM) along the contractile proteins, the subsarcolemmal mitochondria (SM) just beneath the sarcolemmal and perinuclear mitochondria (PN) around the nucleus. m, myofibrils; n, nucleus.

Adult cardiomyocytes are characterized by a complex cytoarchitecture which allows an efficient and synchronized contraction of the entire cell. The maintenance of this cytoarchitecture is ensured by the cytoskeleton which holds sarcomeres in lateral register, mechanically couples myofibrils of adjacent myocytes, and transduces mechanical stress signals. Mitochondria are an integral part of this cytoarchitecture; different populations of those organelles are usually defined according to their location: intermyofibrillar mitochondria, subsarcolemmal mitochondria and perinuclear mitochondria. Those mitochondria are easily identifiable by electron microscopy (Figure 1). Intermyofibrillar mitochondria are strictly ordered between rows of contractile proteins, apparently isolated from each other by repeated arrays of T-tubules, and in close contact with myofibrils and sarcoplasmic reticulum (SR) (Vendelin et al., 2005; Kuznetsov et al., 2009; Guzun et al., 2012); they are mainly devoted to the energy supply of myosin and SR-ATPases. The subsarcolemmal mitochondria present a lower degree of organization and are probably mainly involved in other roles such as ion homeostasis or signaling pathways. Finally, perinuclear mitochondria are organized in clusters and are most probably involved in transcription and translation processes. Concurrently to these functional differences, those mitochondria do not exhibit exactly the same morphology; for example, some authors have shown that intermyofibrillar mitochondria can be larger in certain situations (Ong et al., 2010).

Thus, the mitochondria in muscle cells seem separated from each other and exhibit a unique feature of a highly ordered crystal-like structure which appears optimized for maximal efficacy of energy supply and sustained contraction (Vendelin et al., 2005). Due to spatial constraints and isolation of mitochondria from each other, the energetic regulation of contraction would be confined in the small regions surrounding each sarcomere where the intracellular components interact with each other and compose the ≪Intracellular Energetic Units≫ (ICEU) (Saks et al., 2001; Guzun et al., 2012). The family of creatine kinases (CK), specifically located on the inner-membrane of the mitochondrion, but also at the M-line of myofibrils and on the outer surface of the SR, is an important component of these ICEUs and allows efficient local control over adenine nucleotides and fast energy transfer (Joubert et al., 2001b, 2002b; Tepp et al., 2011), thus emphasizing the importance of subcellular organization and compartmentalization of energy transfer (Joubert et al., 2002c; Piquereau et al., 2010).

Another evidence of the importance of cardiac cell architecture is the existence of microdomains of Ca^2+^ at the interface of the organelles. Different authors have shown that the close association between mitochondria and SR provides “hot spots” of very high calcium concentration in the vicinity of mitochondria (Dorn and Maack, 2013). At energetic level, the existence of direct adenine nucleotide canalization (DANC) (Kaasik et al., 2001) has also been shown to represent a pathway complementary to phosphotransfer enzymes to directly transfer and regulate ATP and ADP levels in microdomains at the interface of the organelles. Interestingly, this cytoarchitecture develops at the time of early cardiac ontogenic development (Piquereau et al., 2010), and when disturbed, the efficiency of this direct canalization is decreased (Wilding et al., 2006). Moreover, a change in osmotic pressure which can induce swelling or shrinkage of mitochondria can modulate the function of other compartments (Kaasik et al., 2004, 2010). Any change of cell architecture, and particularly of mitochondrial morphology, can therefore impact mitochondrial function and energy transfer (Joubert et al., 2008; Piquereau et al., 2012). This is why many authors address the question of how mitochondrial dynamics play a regulatory role in energy production and cell signaling in the heart.

## Mitochondrial dynamics proteins in the heart

Mitochondria are dynamic organelles able to change their morphology in response to different signals. Phenomena that govern mitochondrial morphology aroused real interest since the 1970s when the first fusion events have been described (Kimberg and Loeb, 1972; Wakabayashi et al., 1975; Wakabayashi and Green, 1977). For the last four decades, the mechanistic knowledge of mitochondrial dynamics has been well-developed and, even if some phenomena involved in mitochondrial fusion and fission remain shrouded in mystery, the major proteins governing these processes have been identified. The complete protein machinery of mitochondrial fusion and fission exists in cardiac cells and specifically regulates mitochondrial morphology and size. The currently identified of such proteins and their possible roles in the adult heart are summarized next.

### Proteins involved in fusion and fission of mitochondria

All studies agree so far that fusion of the outer and inner mitochondrial membranes occurs separately and involves distinct molecules. The outer membrane fusion is governed by mitofusins (Mfn1 and Mfn2) (Santel and Fuller, 2001; Legros et al., 2002) while the inner membrane fusion involves Opa1 (Alexander et al., 2000). Interestingly, these two kinds of proteins (Mfns and Opa1) exhibit strong similarities in their structure and their mode of action. Indeed, these proteins contain a GTPase domain, a transmembrane domain allowing the anchorage of the proteins to the outer (Mfns) or inner (Opa1) membranes, and a coiled-coil domain. Whereas their GTPase domain implies a GTP-dependence of the phenomena involved in the mitochondrial fusion (Chen et al., 2003; Ishihara et al., 2004; Olichon et al., 2007), the coiled-coil domain also plays a major role since it allows homotypic (Mfn1-Mfn1, Mfn2-Mfn2 et Opa1-Opa1) or heterotypic (Mfn1-Mfn2) interaction of these proteins (Chen et al., 2003). The non-transmembrane part of Mfns, which is located in the cytosol, allows, through the interaction of the coiled-coil domains of two distinct Mfn proteins, the formation of a physical link between the outer membranes of two neighboring mitochondria (Koshiba et al., 2004; Chan, 2006). Thus, these two outer membranes become closer and the fusion of the involved mitochondria is initiated. In a similar way, the formation of Opa1-Opa1 homotypic complexes leads to the fusion of the inner membranes of the mitochondria engaged in the fusion process (for review see Liesa et al., 2009).

The specificity of action of these proteins is partly due to their anchorage to the membranes of which they control the fusion. However, the specific localization of each protein does not seem to be exclusive since Opa1 exists in a soluble form, which has been detected in an isolated outer mitochondrial membrane fraction, indicating that this Opa1 isoform is able to interact with the outer membrane (Satoh et al., 2003). Besides, this observation combined to the description of a direct interaction between Mfns and Opa1 (Guillery et al., 2008) could in part explain the synchronization of Mfns and Opa1 action. Indeed, the coordination of these proteins is still mysterious because no clear mechanism has been claimed and no equivalent to Ugo1, the yeast protein known to be involved in the coordination of Mfns and Opa1 yeast orthologs (Fzo et Mgm1, Hales and Fuller, 1997; Alexander et al., 2000), has been described in mammals.

Whereas the mechanisms involved in mitochondrial fusion seem to be relatively well-understood, the processes regulating mitochondrial fission still raise some questions. During the end of the 1990s and the beginning of the 2000s, many studies were interested in the dynamin-related protein 1 (Drp1), also called Dynamin-like protein 1 (Dlp1), and the mitochondrial fission protein 1 (Fis1), which were considered as the major actors of mitochondrial fission. Drp1 is a cytosolic protein which comprises a GTPase domain and migrates to mitochondria using dynein and the microtubule (Varadi et al., 2004) or the actine network (De Vos et al., 2005), depending on the fission initiating factor. After this migration, Drp1 is specifically found at the level of the future fission site where it oligomerizes to form a ring, the GTP-dependence constriction of this ring leading to mitochondrial division (Yoon et al., 2001; Ingerman et al., 2005). Interestingly, while phosphorylated Drp1 stays inactive in the cytosol, Drp1 activity can be modulated by calcium through the activation of calcineurin which participates in Drp1 mitochondrial recruitment by dephosphorylating it. This mechanism could be of high significance in muscle cells where calcium is cyclically released from the SR (Cribbs and Strack, 2007; Cereghetti et al., 2008). Knowing that this protein does not exhibit any transmembrane domain necessary for its anchorage to the mitochondrial membranes, its mitochondrial localization requires a docking receptor on the outer mitochondrial membrane. Fis1 was the first protein described as the mitochondrial receptor (Yoon et al., 2003). Fis1 which is anchored in the outer mitochondrial membrane has an intracellular facing domain containing five α-helices which allow oligomerization (for the first α-helix) and direct or indirect interaction with Drp1 (for the all set of α-helices) (Jofuku et al., 2005).

Although many experimental studies have established an undeniable role of Fis1 in mitochondrial fission, the recruitment of Drp1 to mitochondria is not affected by Fis1 deficiency (Lee et al., 2004; Wasiak et al., 2007). Thus, Fis1 does not appear to be the only factor involved in Drp1 mitochondrial recruitment. Three other proteins have been described like the mitochondria fission factor (Mff) (Otera et al., 2010), the mitochondrial dynamics protein of 49 kDa (MiD49) or 51 kDa (MiD51) (Palmer et al., 2011). As Fis1, these three proteins are anchored to the outer mitochondrial membrane (Gandre-Babbe and van der Bliek, 2008; Palmer et al., 2011). The involvement of each protein in mitochondrial fission is, however, not yet clearly defined, and the interaction of these proteins with Drp1 could require other intermediary proteins. This is the case in the yeast in which the interaction of Drp1 and Fis1 orthologs needs several other proteins like Mdv1 and Caf4 (Tieu et al., 2002; Griffin et al., 2005); however, no such proteins have been described in mammals so far.

If a specific machinery of the inner mitochondrial membrane fission exists, it remains unknown. Although, it is suggested that the MDM33 protein is involved in the fission of the inner membrane of *Saccharomyces cerevisae* mitochondria (Messerschmitt et al., 2003), no MDM33 ortholog has been found in mammals. Even if a study proposes that a protein called Mtp18 (Mitochondrial protein 18) could be an actor of this unknown inner mitochondrial membrane fission machinery (Tondera et al., 2005), this clearly requires further investigations.

Finally, mechanisms governing mitochondrial fragmentation seem to be less specific than those involved in mitochondrial fusion. Indeed, Drp1, Fis1, and Mff which are responsible for mitochondrial fission are also implicated in peroxisome fission (Koch et al., 2003, 2005; Gandre-Babbe and van der Bliek, 2008); however, the synchronization or the joint regulation of these two phenomena has never been explored. The fact that the fission of these two organelles involves the same proteins should not be coincidental. It can be noticed that mutations in genes encoding these proteins have been involved in serious diseases, and in particular in neurological diseases such as Charcot-Marie-Tooth type 2A or autosomal dominant optic atrophy (ADOA) (Alexander et al., 2000; Delettre et al., 2001; Zuchner et al., 2004). In heart failure (HF), recent data also suggest their possible implication in the progression of the pathology (Chen et al., 2009), suggesting a role of these proteins in cardiac tissue.

### What about mitochondrial dynamics in the heart?

Whereas it has long been suggested that adult cardiomyocytes would show a limited mitochondrial dynamics because of the complex cytoarchitecture of this cell, the high expression level of dynamin proteins in the heart (Alexander et al., 2000; Delettre et al., 2001; Santel et al., 2003; Gandre-Babbe and van der Bliek, 2008) implies that these actors could play roles which would not be anecdotal. Thus, many research groups have tried to explain mitochondrial dynamics in the heart for a few years. However, knowing that the initial experiments were done with immortalized cardiac cell lines (H9c2, HL-1) or with neonatal cardiomyocytes (Shen et al., 2007; Parra et al., 2008; Twig et al., 2008a) in which mitochondria face an environment very different from the adult one (Leu et al., 2001; Piquereau et al., 2010), the first data about mitochondrial fusion and fission obtained in the mature heart are relatively new and consequently these phenomena are not clearly understood yet.

#### Existence of mitochondrial dynamics in the adult heart

Mitochondrial dynamics comprises two main notions, one is the capacity of mitochondrial to move within the cell and the second relates to the capacity to undergo fusion and fission, these two notions not being mutually exclusive. As presented above, the adult cardiac muscle cell is an extremely organized cell in which the mitochondrial movements are greatly restricted (Beraud et al., 2009; Hom and Sheu, 2009). Moreover, the fusion/fission events appear to be greatly slowed compared to neonatal cardiomyocytes, and mitochondria are poorly connected. It has been recently suggested that the fusion/fission cycle would last 14–16 days in adult cardiomyocytes (Chen et al., 2011). Thus, mitochondrial dynamics could seem irrelevant, although mitochondria have a limited life span, being subjected to biogenesis and autophagy/mitophagy, which are strictly dependent on fusion and fission phenomena (Diaz and Moraes, 2008; Twig et al., 2008b). Consequently, even if fusion or fission events have never been observed in real time, this mitochondrial turnover imposes mitochondrial dynamics as an essential cog of cardiac physiology.

Phenotypic examination of genetically modified mice has recently substantiated our knowledge. Major changes in mitochondrial morphology have been described in mice with inducible cardiac Mfn2 ablation (Papanicolaou et al., 2011), or decrease in Opa1 protein content (Piquereau et al., 2012). However, the observation of larger mitochondria in Mfn2 and Opa1 deficient mice made by Walsh's group (Papanicolaou et al., 2011) and our team (Piquereau et al., 2012) is surprising because it contradicts the previously published data. Indeed, while several studies on non-cardiac cells (Chen et al., 2003; Olichon et al., 2003; Yoon et al., 2003; Cipolat et al., 2004; Stojanovski et al., 2004; Griparic et al., 2007) evidenced that a decrease in expression of a fusion protein would lead, respectively, to mitochondrial network fragmentation, in these animal studies, deficiency in the fusion proteins Mfn2 or Opa1 led to paradoxically larger cardiac mitochondria. Thus, it appears that the specific architectural organization of this cell impact on the phenomena and presumably affects the mode of action of these proteins. On the other hand, the cardiac-specific Mfn1-null mice showed a fragmentation of the mitochondrial network (Papanicolaou et al., 2012); this observation reinforces the idea that Mfn1 and Mfn2 play different roles. Finally, the conditional combined Mfn1/Mfn2 ablation in adult hearts induces mitochondrial network fragmentation (Chen et al., 2011). Thus, mitochondrial dynamics exists in the heart tissue, but is a complex process that depends on the specific cell architecture.

Beyond the fact that these genetically-manipulated mice show obvious changes in cardiac mitochondrial morphology, significant deleterious consequences on their cardiac function were observed under stress (Papanicolaou et al., 2011; Piquereau et al., 2012), suggesting a direct involvement of the mitochondrial morphology in the heart function. At present, it is complicated to determine how and to which extent the morphology of mitochondria affects cardiac efficiency. For example, our group has already evidenced that changes in the mitochondrial volume may directly impact the force developed by myofibrils (Kaasik et al., 2004) as well as the direct energetic transfers between mitochondria and myofilaments (Piquereau et al., 2012). Even if these studies are insufficient to conclude that these mechanisms are responsible for the cardiac alterations observed in the previously described models, they suggest a direct link between mitochondrial morphology and cardiac contractile function. However, it is not easy to get an overall understanding of the mechanisms governing mitochondrial fusion and fission in the heart, even if it obviously seems that mitochondrial dynamics exists in this organ on a very slow time-course in the normal heart.

#### Impact of mitochondrial dynamics alterations on respiratory capacities

It is generally accepted that fusion-fission processes impact on the mitochondrial energetics in cultured cells (Chen et al., 2003). Disruption of mitochondrial dynamics by overexpression or suppression of fusion (Mfn2, Opa1) (Olichon et al., 2003; Chen et al., 2005) or mitochondrial fission (Benard et al., 2007), can cause alterations in mitochondrial metabolism (Chen and Chan, 2005), according to the degree of differentiation of the considered cell type. These changes may be accompanied by the modulation of the mitochondrial membrane potential, and of the expression of complex I, IV, and V subunits (Pich et al., 2005; Chan, 2006; Liesa et al., 2009). However, in patients and in mouse models, direct effect of dynamin mutations on mitochondrial function gave conflicting results, probably because of the diversity of the cell types and the mutations studied (Olichon et al., 2006). In general, it is considered that fragmentation induced by an increase in Drp1 or a decrease in Mfn2 or Opa1 is harmful and leads to metabolic disorders (Parra et al., 2011). In contrast, fusion is generally considered rather beneficial. But a careful look at the literature shows that there is no clear link between mitochondria morphology and respiratory capacities, especially in mature cardiac cell. Indeed, in three different studies, no obvious alterations of respiratory chain function were observed when Opa1, Mfn1 or Mfn2 were separately genetically downregulated (Papanicolaou et al., 2011, 2012; Piquereau et al., 2012), except for free fatty-acid utilization in Opa1^+/−^ mice (Piquereau et al., 2012). In another study where a different Opa1 mutation was studied, mitochondrial function alterations were only observed in old mice (Chen et al., 2012). Finally, when complete/double KO (Mfn1/Mfn2) was used to suppress fusion proteins, decreased oxygen consumption or increased oxidative stress was observed (Chen et al., 2011; Dorn et al., 2011). Thus, compensatory mechanisms with other dynamin proteins may exist and attenuate the consequences of one dynamin protein deficiency, but complete loss or aging will be detrimental for mitochondrial function. In addition to an effect on respiratory chain function, other mitochondrial functions can also be affected by mitochondrial dynamics protein alterations.

#### Implication in mitochondrial biogenesis

As mentioned above, the mechanisms involved in mitochondrial dynamics in the cardiomyocyte seem not to be exactly similar to those observed in proliferative cells. However, this complexity is easily understood when the cardiomyocyte is regarded as a cell presenting a strictly organized intracellular architecture which is not in favor of mitochondrial plasticity as in dividing cells. It is thus important to keep in mind this notion when extrapolating results obtained during the early development to adulthood. Obviously, the role of mitochondrial dynamics is different in prenatal and neonatal cardiomyocytes which proliferate and consequently need the synthesis of new mitochondria to assure a suitable repartition of these organelles between daughter cells obtained after mitosis, and in non-dividing adult cardiomyocytes. Not surprisingly, we already described a remarkably high level of expression of mitochondrial biogenesis and dynamics genes during the early stages of postnatal development in the heart (Piquereau et al., 2013). Thus, this important stimulation of mitochondrial biogenesis and therefore of mitochondrial dynamics during development could explain why the cardiac-specific ablation of dynamins during prenatal or early postnatal development quickly leads to HF and death while several months are needed when the ablation occurs at the beginning of adulthood (Chen et al., 2011; Papanicolaou et al., 2012; Dorn, 2013). More generally, this could explain the lethality of the total dynamin knock-out models which die *in utero* (Alavi et al., 2007; Chen et al., 2011), a period during which the mitotic activity is undeniably substantial.

Globally, it would seem that mitochondrial dynamics is particularly important when the heart is under conditions which require the synthesis of new mitochondria. This is the case during development, but also in stress conditions like ischemia (Ong et al., 2010) or pressure overload (Piquereau et al., 2012) or ageing (Chen et al., 2012) which induce increased energetic demand and mitochondrial damages and consequently requires an adequate mitochondrial turnover. That is certainly the reason why the mice genetically manipulated to abolish Mfn2 expression or to decrease Opa1 expression exhibit a high sensitivity to stress. This highlights the fact that the reduced mitochondrial dynamics can be partly compensated under baseline conditions while under stress the mitochondrial turnover cannot be ensured and precipitates cardiac dysfunction. Finally, inducible cardiac-specific double ablation of Mfn1 and Mfn2 genes quickly led to dilated cardiomyopathy before inducing death within 9 weeks after gene ablation. Interestingly, the heterozygous mutation of the Drp1 gene also induced a dilated cardiomyopathy in mice (Ashrafian et al., 2010). The cardiac dysfunction observed in these genetically-manipulated mice clearly shows that these proteins play a fully-fledged role in cardiac muscular cell physiology.

#### Other roles of mitochondrial dynamics proteins

**Figure 2:**
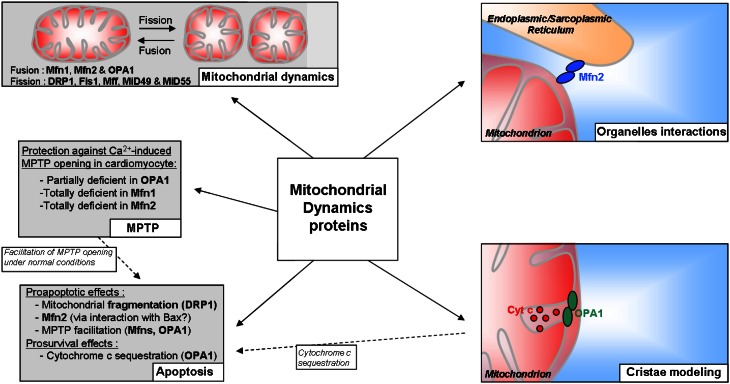
**Scheme summarizing the potential roles of mitochondrial dynamics proteins in the adult cardiomyocyte**.

All dynamins described above are of course widely known for the role they play in mitochondrial dynamics; however, these proteins could have a broader field of action than it seems (Figure 2). Several studies assigned them an effect on the property of the MPTP, a non-selective pore which induces, in particular situations, a high permeability of the mitochondrial membranes and can lead to cell death (Di Lisa and Bernardi, 2009). Indeed, we and others showed that the adult cardiomyocytes partially deficient in Opa1 (Piquereau et al., 2012) or totally deficient in Mfn1 or Mfn2 (Papanicolaou et al., 2011, 2012) exhibit a delayed Ca^2+^-induced MPTP opening. Knowing that these observations have been obtained in isolated cardiomyocytes with enlarged or fragmented mitochondria, they suggest that the individual volume of the mitochondria are not directly involved and that Opa1 and mitofusins could facilitate MPTP opening under normal conditions. The mechanisms leading to this protection are far from being elucidated. Moreover, the data obtained from adult heart deficient in the two mitofusins, which did not show any change in MPTP sensitivity in comparison with wild type (Chen et al., 2011), complicate the understanding even if the experimental conditions differ (isolated mitochondria vs. cardiomyocytes).

Whereas their involvement in the prevention of MPTP opening could indirectly confer to Opa1 and mitofusins an indirect role in cardiomyocyte death, it seems, however, that the role of the mitochondrial dynamics proteins in cell death is not really clear. It is well-known that mitochondria are central elements in cell death, and a link between mitochondrial network fragmentation and apoptosis has been already described in cell lines (Frank et al., 2001; Breckenridge et al., 2003) and in neonatal cardiomyocytes, suggesting an important role of fission protein, especially Drp1 (Parra et al., 2008; Wakabayashi et al., 2009), in this form of cell death. The fusion proteins could also be directly involved in cell death by acting in an original way. Even if the data obtained from studies realized with cell lines assert that fusion is prosurvival while fission is proaptotic, Mfn2 could participate in apoptosis. The participation of Mfn2 to this particular death has been demonstrated in cardiomyocytes and smooth muscle cells (Guo et al., 2007; Shen et al., 2007). The details of this mechanism are still unclear, although an interaction between Mfn2 and the pro-apoptotic protein Bax has been described (Hoppins et al., 2011b). On the other hand, Opa1 would have an opposite role and could be an antiapoptotic factor. Indeed, this protein would regulate cell death by forming oligomers of two Opa1 proteins creating a “bottle neck”-like structure which allows cytochrome c sequestration in the cristae (Frezza et al., 2006). By this oligomerization, Opa1 is in fact a crucial protein in the mitochondrial internal organization which is, as stated above, essential for the functions of these organelles.

Thus, mitochondrial dynamics proteins are clearly essential for the adaptation of those organelles to cell status. Moreover, some of them could be at the heart of the interaction between mitochondria and other components of the cell. Indeed, Mfn2, but not Mfn1, is also found at the endoplasmic reticulum (ER) membrane and thus can create physical links between ER and mitochondria (de Brito and Scorrano, 2008). This link has been recently shown between mitochondria and SR in muscle cells (Dorn and Maack, 2013); considering the calcium and energetic microdomains at the interface of mitochondria and SR described in the cardiomyocytes, Mfn2 could be a major actor of the contractile function of the heart.

Moreover, the physical links between ER and mitochondria were demonstrated to increase following ER stress (Csordas et al., 2006), a specific response triggered when ER homeostasis is disrupted and ER function is compromised. In the heart, ER stress has recently been recognized as an important contributor to the development of cardiac dysfunction (Groenendyk et al., 2013), and the link between ER stress and Mfns has started to be investigated. By using knock-out mice, Ngoh and colleagues demonstrated that cardiomyocyte-specific deletion of Mfn2, but not Mfn1, induces ER stress *in vivo*, leading to the conclusion that Mfn2 is a negative regulator of ER stress required for the homeostasis of the ER (Ngoh et al., 2012).

Even if the mechanism is not fully understood, Mfn2 is also involved in cardiac autophagic processes (Zhao et al., 2012). These authors showed that Mfn2 could participate in autophagosome-lysosome fusion. Due to the presence of Mfn2 in the ER and thus autophagosome membrane, it might act as an adaptor protein mediating autophagosome maturation. This link between Mfn2 and autophagy is reminiscent of the fact that mitochondrial dynamics proteins are also major actors of selective mitochondrial autophagy, i.e., mitophagy. Indeed, it is largely admitted that selective clearance of mitochondria are preceded by fission phenomena (Twig et al., 2008b). In fact, in the early event of mitophagy, Drp1 is recruited to mitochondria (Lee et al., 2011) and Mfns, present at the membrane of damaged mitochondria, are rapidly ubiquitined (Ziviani et al., 2010). This Mfns ubiquitination could thus address these proteins to the proteasome or could interfere with mitochondrial tethering and prevent altered mitochondria to join the mitochondria network (Ziviani and Whitworth, 2010). Besides, in addition to the low mitochondrial potential allowing identification of mitochondria destined to mitophagy, these mitochondria would exhibit a low Opa1 amount at the inner membrane (Twig et al., 2008a). This creates non-fusing mitochondria which thus have only one destiny: degradation.

Finally, changes in the components of mitochondrial dynamics can also alter mtDNA. Observations in S. cerevisiae and MEF cells indicate that the normal activity of Opa1 (Jones and Fangman, 1992; Guan et al., 1993; Chen et al., 2007) or Mfns (Hermann et al., 1998; Rappaport et al., 1998; Chen et al., 2003, 2007) are crucial for maintaining the integrity of mtDNA nucleoids (Liesa et al., 2009). Part of Opa1 could be involved in the attachment of mtDNA to the inner mitochondrial membrane and to promote mtDNA replication and distribution (Elachouri et al., 2011). In the mature heart, one study reported that heterozygous Opa1^+/−^ mice exhibit reduced mtDNA copy number (Chen et al., 2012), which could be involved in the development of cardiac mitochondrial dysfunction.

Finally, even if the number of studies devoted to the role of mitochondrial dynamics proteins in the heart is limited (Table 1), it can be asserted that these proteins are clearly integral part of cardiomyocyte life by ensuring fusion/fission processes and by participating in the several mechanisms described above. Interestingly, it seems that these proteins would be particularly important under stress conditions which are known to mobilize or affect mitochondrial functions. These kinds of situations exacerbate mitochondrial biogenesis and turnover which are relatively low under basal conditions, showing the major significance of the processes governed by these proteins in cardiac adaptations.

**Table 1:** **Mitochondrial dynamic studies on the mature heart**.

**Authors**	**Tissue**	**Model**	**Mitochondrial morphology**	**Alterations**	**Observations**
Shahrestani et al., 2009	Drosophila	OPA1^+/−^		Decreased heart rate and increased heart arrhythmia	Poor tolerance to stress induced by electrical pacing
Dorn et al., 2011	Drosophila	OPA1 RNAi	Decrease of mean mitochondrial size	Contractile abnormality and remodeling	Stimulation of mitochondrial biogenesis
Piquereau et al., 2012	Mouse (3–6 months)	OPA1^+/−^	Enlarged mitochondria, cristae disorganization	No alteration of cardiac function nor change in QO_2_, but delay of MPTP opening and energy transfer alteration	More sensitive to transaortic constriction (TAC)
Chen et al., 2012	Mouse (12 months)	OPA1^+/−^	Disorganization of mitochondrial network	Reduced mtDNA level, mitochondria and cell dysfunction	Increased of oxidative stress and late-onset cardiomyopathy
Dorn et al., 2011	Drosophila	Marf RNAi	Decrease of mean mitochondrial size	Contractile abnormality and remodeling	Stimulation of mitochondrial biogenesis
Papanicolaou et al., 2011	Mouse	Mfn2 KO	Enlarged mitochondria	No major cardiac and mitochondrial dysfunction, delay of MPTP opening	Protection against cell death induced injury and better recovery after I/R
Papanicolaou et al., 2012	Mouse	Mfn1 KO	Fragmented mitochondria	Normal cardiac and mitochondrial function, decreased of ROS induced MPTP opening	protection against ROS induced mitochondrial dysfunction
Chen et al., 2012	Mouse	Mfn1/Mfn2 DKO	Fragmented mitochondria	Cardiomyocyte and mitochondrial respiratory dysfunction	Progressive and lethal dilated cardiomyopathy
Ngoh et al., 2012	mice	Inducible Mfn2 KO	Fragmentation	Increased markers of the ER stress	Mfn2 is necessary for ER homeostasis
Ashrafian et al., 2010	Mouse	Drp1 mutation^+/−^		Reduced mitochondrial complexes levels and cardiac ATP depletion	Energy deficiency may contribute to cardiomyopathy
Chen et al., 2009	Rat	Heart failure	Decrease of mean individual mitochondrial size	decrease of OPA1 protein level	
Ong et al., 2010	Rat	Ischemia/reperfusion and drp1 inhibition by mdivi-1	Presence of elongated mitochondria in control heart	Mitochondrial fragmentation prevented by mdivi-1 after I/R	Decrease of infarct size in I/R after treatment with an inhibitor of Drp1

## Alterations of mitochondrial dynamics in cardiac pathologies and possible therapeutic approaches

Chronic HF is associated with morphologic abnormalities of cardiac mitochondria including increased number, reduced organelles size, and compromised structural integrity (Schaper et al., 1991; Sabbah et al., 1992; Beutner et al., 2001), suggesting fragmentation of the mitochondrial network (Joubert et al., 2008). Mitochondrial damages as the depletion of the mitochondrial matrix and disruption of membranes positively correlate with the HF severity index (Sabbah et al., 1992) and it is recognized that mitochondria can determine the cellular fate (Di Lisa and Bernardi, 1998). In other pathologies, mega-mitochondria can appear (for review, see Wakabayashi, 2002 and Hoppel et al., 2009). Most of the time, heterogeneity of the size and distribution of cardiac mitochondria increases in HF, evidencing unbalanced fusion/fission cycles. Thus, an emerging hypothesis is that the mechanisms that control the shape of mitochondria may play a role in cardiac pathologies. In particular, a recent study suggested that Opa1 could be downregulated in HF (Chen et al., 2009; Chen and Knowlton, 2010). Another study observed a decrease in Mfn2, an increase in Fis1, and no change in Opa1 expression in rat hearts 12–18 weeks after myocardial infarction (Javadov et al., 2011). However, alteration of dynamin proteins could merely be the consequence of the alteration of mitochondrial biogenesis as it has been suggested (Garnier et al., 2005; Ventura-Clapier et al., 2011). Indeed, a strict relation exists between the PPAR gamma coactivator-1 (PGC-1α), a master regulator of mitochondrial biogenesis, and the expression of these proteins (Garnier et al., 2005). Moreover, in most cardiac pathologies where defect in dynamin proteins are observed, a decrease in mitochondrial mass is also present (Ventura-Clapier et al., 2011; Parra et al., 2011). So it is not clear so far which protein alteration is responsible for a possible unbalance in fusion/fission, and the post-translational modifications of, for example, Drp1 (see above) could also be involved. Similarly, nothing is known about a possible implication of GTP/GDP supply in mitochondrial dynamics regulation.

Cell death is also an important pathophysiological process in both HF and in cardiac ischemia. However, the underlying mechanisms by which the heart looses myocytes in HF are not completely understood. Mitochondria have a critical role in regulating cardiac cell death. If fission is interrupted, large networks of fused mitochondria occur. If fusion fails, mitochondria become smaller and fragmented. Abnormalities in fission and fusion can lead to apoptosis (Lee et al., 2004; Cassidy-Stone et al., 2008) which is an important mechanism of cardiac myocyte loss in HF (Olivetti et al., 1997; Narula et al., 1999). So both HF and ischemia could be associated with abnormalities of fission and fusion that would contribute to cardiac cell death and change of MPTP sensitivity. In particular, after ischemia-reperfusion, mitochondria of cardiomyocytes show heterogeneous damages in their morphology, in redox status and in calcium homeostasis, which could be related to an overproduction of local ROS (Ong et al., 2012).

Modulation of mitochondrial dynamics appears as a novel pharmacological strategy for cardioprotection, in particular to protect the heart after a heart attack, and in ischemia-reperfusion [see reviews by Ong et al. (2012) and Dorn (2013)]. Several studies have shown that changes in mitochondrial morphology, including targeting proteins of the mitochondrial dynamics could allow the heart to better recover from an ischemic insult (Ong et al., 2010; Papanicolaou et al., 2011). In the first study, the authors used a specific inhibitor of Drp1, Mdiv-1, to prevent mitochondrial fission, and observed a significant reduction in myocardial infarct size in the *in vivo* murine heart. In the second one, using a genetic model of Mfn2 KO mice, they observed an improvement of cardiac performance following *ex vivo* ischemia/reperfusion. In both cases, even if the strategy was different (decrease the fission in one case, decrease the fusion in the second case), they linked the protection to the inhibition of MPTP opening, which is a classical target of cardioprotection. However, it should be kept in mind that although inhibition of MPTP can be beneficial in short term, it could be detrimental on the long term (Elrod et al., 2010; Piquereau et al., 2012).

## Conclusion

Generally, studies described above prove that mitochondrial dynamics proteins are necessary for normal mitochondrial functions in the cardiomyocyte. In this cell where the internal organization is an obstacle to organelles mobility, these proteins govern slow fusion/fission processes which ensure mitochondrial turnover required to maintain mitochondrial function and consequently organ function. It also seems that mitochondrial dynamics would be a key element of cardiomyocyte adaptation under stress which induce alteration of mitochondria and thus lead to generation of new mitochondria and degradation of damaged mitochondria. Under such conditions, mitochondrial dynamics processes would be exacerbated and would assume greater significance, explaining why the genetically-manipulated mice for mitochondrial dynamics genes are particularly sensitive to stress. Therefore, the high expression level of dynamins under basal conditions, despite the slow fusion/fission cycle, could be a major element of cardiac adaptation. In addition, those proteins could be activated by post-translational modifications involved in signaling pathways activated in response to stress. It can be anticipated that those potential post-translational modifications would be the more efficient way to ensure an optimal reactivity of mitochondrial dynamics machinery under stress.

Finally, mitochondrial dynamics proteins are involved in several phenomena irrespectively of their role in mitochondrial dynamics. This aspect of these dynamins has to be kept in mind because they are essential for the adequate function of mitochondria and cell life. By their extended field of action, these proteins are clearly established as major components of cardiac physiology.

### Conflict of interest statement

The authors declare that the research was conducted in the absence of any commercial or financial relationships that could be construed as a potential conflict of interest.

## References

[B1] Alavi M. V. Bette S. Schimpf S. Schuettauf F. Schraermeyer U. Wehrl H. F. (2007). A splice site mutation in the murine Opa1 gene features pathology of autosomal dominant optic atrophy. Brain 130, 1029–1042 10.1093/brain/awm005 17314202

[B2] Alexander C. Votruba M. Pesch U. E. Thiselton D. L. Mayer S. Moore A. (2000). OPA1, encoding a dynamin-related GTPase, is mutated in autosomal dominant optic atrophy linked to chromosome 3q28. Nat. Genet. 26, 211–215 10.1038/79944 11017080

[B3] Ashrafian H. Docherty L. Leo V. Towlson C. Neilan M. Steeples V. (2010). A mutation in the mitochondrial fission gene Dnm1l leads to cardiomyopathy. PLoS Genet. 6:e1001000 10.1371/journal.pgen.1001000 20585624 PMC2891719

[B4] Balaban R. S. (2012). Perspectives on: SGP symposium on mitochondrial physiology and medicine: metabolic homeostasis of the heart. J. Gen. Physiol. 139, 407–414 10.1085/jgp.201210783 22641635 PMC3362523

[B5] Benard G. Bellance N. James D. Parrone P. Fernandez H. Letellier T. (2007). Mitochondrial bioenergetics and structural network organization. J. Cell Sci. 120, 838–848 10.1242/jcs.03381 17298981

[B6] Beraud N. Pelloux S. Usson Y. Kuznetsov A. V. Ronot X. Tourneur Y. (2009). Mitochondrial dynamics in heart cells: very low amplitude high frequency fluctuations in adult cardiomyocytes and flow motion in non beating Hl-1 cells. J. Bioenerg. Biomembr. 41, 195–214 10.1007/s10863-009-9214-x 19399598

[B7] Bereiter-Hahn J. (1990). Behavior of mitochondria in the living cell. Int. Rev. Cytol. 122, 1–63 2246114 10.1016/s0074-7696(08)61205-x

[B8] Beutner G. Sharma V. K. Giovannucci D. R. Yule D. I. Sheu S. S. (2001). Identification of a ryanodine receptor in rat heart mitochondria. J. Biol. Chem. 276, 21482–21488 10.1074/jbc.M101486200 11297554

[B9] Breckenridge D. G. Stojanovic M. Marcellus R. C. Shore G. C. (2003). Caspase cleavage product of BAP31 induces mitochondrial fission through endoplasmic reticulum calcium signals, enhancing cytochrome c release to the cytosol. J. Cell Biol. 160, 1115–1127 10.1083/jcb.200212059 12668660 PMC2172754

[B10] Campello S. Scorrano L. (2010). Mitochondrial shape changes: orchestrating cell pathophysiology. EMBO Rep. 11, 678–684 10.1038/embor.2010.115 20725092 PMC2933866

[B11] Cassidy-Stone A. Chipuk J. E. Ingerman E. Song C. Yoo C. Kuwana T. (2008). Chemical inhibition of the mitochondrial division dynamin reveals its role in Bax/Bak-dependent mitochondrial outer membrane permeabilization. Dev. Cell 14, 193–204 10.1016/j.devcel.2007.11.019 18267088 PMC2267902

[B12] Cereghetti G. M. Stangherlin A. Martins de Brito O. Chang C. R. Blackstone C. Bernardi P. (2008). Dephosphorylation by calcineurin regulates translocation of Drp1 to mitochondria. Proc. Natl. Acad. Sci. U.S.A. 105, 15803–15808 10.1073/pnas.0808249105 18838687 PMC2572940

[B13] Chan D. C. (2006). Mitochondrial fusion and fission in mammals. Annu. Rev. Cell Dev. Biol. 22, 79–99 10.1146/annurev.cellbio.22.010305.104638 16704336

[B14] Chen H. Chan D. C. (2005). Emerging functions of mammalian mitochondrial fusion and fission. Hum. Mol. Genet. 14, R283–R289 10.1093/hmg/ddi270 16244327

[B15] Chen H. Chomyn A. Chan D. C. (2005). Disruption of fusion results in mitochondrial heterogeneity and dysfunction. J. Biol. Chem. 280, 26185–26192 10.1074/jbc.M503062200 15899901

[B16] Chen H. Detmer S. A. Ewald A. J. Griffin E. E. Fraser S. E. Chan D. C. (2003). Mitofusins Mfn1 and Mfn2 coordinately regulate mitochondrial fusion and are essential for embryonic development. J. Cell Biol. 160, 189–200 10.1083/jcb.200211046 12527753 PMC2172648

[B17] Chen H. McCaffery J. M. Chan D. C. (2007). Mitochondrial fusion protects against neurodegeneration in the cerebellum. Cell 130, 548–562 10.1016/j.cell.2007.06.026 17693261

[B18] Chen L. Gong Q. Stice J. P. Knowlton A. A. (2009). Mitochondrial OPA1, apoptosis, and heart failure. Cardiovasc. Res. 84, 91–99 10.1093/cvr/cvp181 19493956 PMC2741347

[B19] Chen L. Knowlton A. A. (2010). Mitochondria and heart failure: new insights into an energetic problem. Minerva Cardioangiol. 58, 213–229 20440251 PMC3786553

[B20] Chen L. Liu T. Tran A. Lu X. Tomilov A. A. Davies V. (2012). OPA1 mutation and late-onset cardiomyopathy: mitochondrial dysfunction and mtDNA instability. J. Am. Heart Assoc. 1:e003012 10.1161/JAHA.112.003012 23316298 PMC3541627

[B21] Chen Y. Liu Y. Dorn G. W. 2nd. (2011). Mitochondrial fusion is essential for organelle function and cardiac homeostasis. Circ. Res. 109, 1327–1331 10.1161/CIRCRESAHA.111.258723 22052916 PMC3237902

[B22] Cipolat S. Martins de Brito O. Dal Zilio B. Scorrano L. (2004). OPA1 requires mitofusin 1 to promote mitochondrial fusion. Proc. Natl. Acad. Sci. U.S.A. 101, 15927–15932 10.1073/pnas.0407043101 15509649 PMC528769

[B23] Collins T. J. Berridge M. J. Lipp P. Bootman M. D. (2002). Mitochondria are morphologically and functionally heterogeneous within cells. EMBO J. 21, 1616–1627 10.1093/emboj/21.7.1616 11927546 PMC125942

[B24] Cribbs J. T. Strack S. (2007). Reversible phosphorylation of Drp1 by cyclic AMP-dependent protein kinase and calcineurin regulates mitochondrial fission and cell death. EMBO Rep. 8, 939–944 10.1038/sj.embor.7401062 17721437 PMC2002551

[B25] Csordas G. Renken C. Varnai P. Walter L. Weaver D. Buttle K. F. (2006). Structural and functional features and significance of the physical linkage between ER and mitochondria. J. Cell Biol. 174, 915–921 10.1083/jcb.200604016 16982799 PMC2064383

[B26] Davies K. M. Daum B. Kuhlbrandt W. Anselmi C. Faraldo-Gomez J. (2012). Structure of the mitochondrial ATP synthase and its role in shaping mitochondria cristae. Microsc. Microanal. 18 Suppl. 2, 56–57 10.1017/S143192761200213923177446

[B27] Davies K. M. Strauss M. Daum B. Kief J. H. Osiewacz H. D. Rycovska A. (2011). Macromolecular organization of ATP synthase and complex I in whole mitochondria. Proc. Natl. Acad. Sci. U.S.A. 108, 14121–14126 10.1073/pnas.1103621108 21836051 PMC3161574

[B28] de Brito O. M. Scorrano L. (2008). Mitofusin 2 tethers endoplasmic reticulum to mitochondria. Nature 456, 605–610 10.1038/nature07534 19052620

[B29] Delettre C. Griffoin J. M. Kaplan J. Dollfus H. Lorenz B. Faivre L. (2001). Mutation spectrum and splicing variants in the OPA1 gene. Hum. Genet. 109, 584–591 10.1007/s00439-001-0633-y 11810270

[B30] De Vos K. J. Allan V. J. Grierson A. J. Sheetz M. P. (2005). Mitochondrial function and actin regulate dynamin-related protein 1-dependent mitochondrial fission. Curr. Biol. 15, 678–683 10.1016/j.cub.2005.02.064 15823542

[B31] Diaz F. Moraes C. T. (2008). Mitochondrial biogenesis and turnover. Cell Calcium 44, 24–35 10.1016/j.ceca.2007.12.004 18395251 PMC3175594

[B32] Di Lisa F. Bernardi P. (1998). Mitochondrial function as a determinant of recovery or death in cell response to injury. Mol. Cell Biochem. 184, 379–391 9746332

[B33] Di Lisa F. Bernardi P. (2009). A CaPful of mechanisms regulating the mitochondrial permeability transition. J. Mol. Cell Cardiol. 46, 775–780 10.1016/j.yjmcc.2009.03.006 19303419

[B34] Dorn G. W. 2nd. (2013). Mitochondrial dynamics in heart disease. Biochim. Biophys. Acta 1833, 233–241 10.1016/j.bbamcr.2012.03.008 22450031 PMC3390438

[B35] Dorn G. W. 2nd. Clark C. F. Eschenbacher W. H. Kang M. Y. Engelhard J. T. Warner S. J. (2011). MARF and Opa1 control mitochondrial and cardiac function in Drosophila. Circ. Res. 108, 12–17 10.1161/CIRCRESAHA.110.236745 21148429 PMC3337031

[B36] Dorn G. W. 2nd. Maack C. (2013). SR and mitochondria: calcium cross-talk between kissing cousins. J. Mol. Cell Cardiol. 55, 42–49 10.1016/j.yjmcc.2012.07.015 22902320

[B37] Elachouri G. Vidoni S. Zanna C. Pattyn A. Boukhaddaoui H. Gaget K. (2011). OPA1 links human mitochondrial genome maintenance to mtDNA replication and distribution. Genome Res. 21, 12–20 10.1101/gr.108696.110 20974897 PMC3012919

[B38] Elrod J. W. Wong R. Mishra S. Vagnozzi R. J. Sakthievel B. Goonasekera S. A. (2010). Cyclophilin D controls mitochondrial pore-dependent Ca(2+) exchange, metabolic flexibility, and propensity for heart failure in mice. J. Clin. Invest. 120, 3680–3687 10.1172/JCI43171 20890047 PMC2947235

[B39] Frank S. Gaume B. Bergmann-Leitner E. S. Leitner W. W. Robert E. G. Catez F. (2001). The role of dynamin-related protein 1, a mediator of mitochondrial fission, in apoptosis. Dev. Cell 1, 515–525 10.1016/S1534-5807(01)00055-7 11703942

[B40] Frezza C. Cipolat S. Martins de Brito O. Micaroni M. Beznoussenko G. V. Rudka T. (2006). OPA1 controls apoptotic cristae remodeling independently from mitochondrial fusion. Cell 126, 177–189 10.1016/j.cell.2006.06.025 16839885

[B41] Gandre-Babbe S. van der Bliek A. M. (2008). The novel tail-anchored membrane protein Mff controls mitochondrial and peroxisomal fission in mammalian cells. Mol. Biol. Cell 19, 2402–2412 10.1091/mbc.E07-12-1287 18353969 PMC2397315

[B42] Garnier A. Fortin D. Zoll J. N'Guessan B. Mettauer B. Lampert E. (2005). Coordinated changes in mitochondrial function and biogenesis in healthy and diseased human skeletal muscle. FASEB J. 19, 43–52 10.1096/fj.04-2173com 15629894

[B43] Griffin E. E. Graumann J. Chan D. C. (2005). The WD40 protein Caf4p is a component of the mitochondrial fission machinery and recruits Dnm1p to mitochondria. J. Cell Biol. 170, 237–248 10.1083/jcb.200503148 16009724 PMC2171414

[B44] Griparic L. Kanazawa T. van der Bliek A. M. (2007). Regulation of the mitochondrial dynamin-like protein Opa1 by proteolytic cleavage. J. Cell Biol. 178, 757–764 10.1083/jcb.200704112 17709430 PMC2064541

[B45] Groenendyk J. Agellon L. B. Michalak M. (2013). Coping with endoplasmic reticulum stress in the cardiovascular system. Annu. Rev. Physiol. 75, 49–67 10.1146/annurev-physiol-030212-183707 23020580

[B46] Guan K. Farh L. Marshall T. K. Deschenes R. J. (1993). Normal mitochondrial structure and genome maintenance in yeast requires the dynamin-like product of the MGM1 gene. Curr. Genet. 24, 141–148 7916673 10.1007/BF00324678

[B47] Guillery O. Malka F. Landes T. Guillou E. Blackstone C. Lombes A. (2008). Metalloprotease-mediated OPA1 processing is modulated by the mitochondrial membrane potential. Biol. Cell 100, 315–325 10.1042/BC20070110 18076378

[B48] Guo X. Chen K. H. Guo Y. Liao H. Tang J. Xiao R. P. (2007). Mitofusin 2 triggers vascular smooth muscle cell apoptosis via mitochondrial death pathway. Circ. Res. 101, 1113–1122 10.1161/CIRCRESAHA.107.157644 17901359

[B49] Guzun R. Gonzalez-Granillo M. Karu-Varikmaa M. Grichine A. Usson Y. Kaambre T. (2012). Regulation of respiration in muscle cells *in vivo* by VDAC through interaction with the cytoskeleton and MtCK within mitochondrial interactosome. Biochim. Biophys. Acta 1818, 1545–1554 10.1016/j.bbamem.2011.12.034 22244843

[B50] Hackenbrock C. R. (1966). Ultrastructural bases for metabolically linked mechanical activity in mitochondria. I. Reversible ultrastructural changes with change in metabolic steady state in isolated liver mitochondria. J. Cell Biol. 30, 269–297 5968972 10.1083/jcb.30.2.269 PMC2107001

[B51] Hales K. G. Fuller M. T. (1997). Developmentally regulated mitochondrial fusion mediated by a conserved, novel, predicted GTPase. Cell 90, 121–129 10.1016/S0092-8674(00)80319-0 9230308

[B52] Hermann G. J. Thatcher J. W. Mills J. P. Hales K. G. Fuller M. T. Nunnari J. (1998). Mitochondrial fusion in yeast requires the transmembrane GTPase Fzo1p. J. Cell Biol. 143, 359–373 10.1083/jcb.143.2.359 9786948 PMC2132826

[B53] Hinshaw J. E. (2000). Dynamin and its role in membrane fission. Annu. Rev. Cell Dev. Biol. 16, 483–519 10.1146/annurev.cellbio.16.1.483 11031245 PMC4781412

[B54] Hom J. Sheu S. S. (2009). Morphological dynamics of mitochondria–a special emphasis on cardiac muscle cells. J. Mol. Cell Cardiol. 46, 811–820 10.1016/j.yjmcc.2009.02.023 19281816 PMC2995918

[B55] Hoppel C. L. Tandler B. Fujioka H. Riva A. (2009). Dynamic organization of mitochondria in human heart and in myocardial disease. Int. J. Biochem. Cell Biol. 41, 1949–1956 10.1016/j.biocel.2009.05.004 19446651 PMC3221317

[B56] Hoppins S. Collins S. R. Cassidy-Stone A. Hummel E. Devay R. M. Lackner L. L. (2011a). A mitochondrial-focused genetic interaction map reveals a scaffold-like complex required for inner membrane organization in mitochondria. J. Cell Biol. 195, 323–340 10.1083/jcb.201107053 21987634 PMC3198156

[B57] Hoppins S. Edlich F. Cleland M. M. Banerjee S. McCaffery J. M. Youle R. J. (2011b). The soluble form of Bax regulates mitochondrial fusion via MFN2 homotypic complexes. Mol. Cell 41, 150–160 10.1016/j.molcel.2010.11.030 21255726 PMC3072068

[B58] Ingerman E. Perkins E. M. Marino M. Mears J. A. McCaffery J. M. Hinshaw J. E. (2005). Dnm1 forms spirals that are structurally tailored to fit mitochondria. J. Cell Biol. 170, 1021–1027 10.1083/jcb.200506078 16186251 PMC2171542

[B59] Ishihara N. Eura Y. Mihara K. (2004). Mitofusin 1 and 2 play distinct roles in mitochondrial fusion reactions via GTPase activity. J. Cell Sci. 117, 6535–6546 10.1242/jcs.01565 15572413

[B60] Javadov S. Rajapurohitam V. Kilic A. Hunter J. C. Zeidan A. Said Faruq N. (2011). Expression of mitochondrial fusion-fission proteins during post-infarction remodeling: the effect of NHE-1 inhibition. Basic Res. Cardiol. 106, 99–109 10.1007/s00395-010-0122-3 20886221

[B61] Jofuku A. Ishihara N. Mihara K. (2005). Analysis of functional domains of rat mitochondrial Fis1, the mitochondrial fission-stimulating protein. Biochem. Biophys. Res. Commun. 333, 650–659 10.1016/j.bbrc.2005.05.154 15979461

[B62] Jones B. A. Fangman W. L. (1992). Mitochondrial DNA maintenance in yeast requires a protein containing a region related to the GTP-binding domain of dynamin. Genes Dev. 6, 380–389 10.1101/gad.6.3.380 1532158

[B63] Joubert F. Gillet B. Mazet J. L. Mateo P. Beloeil J. Hoerter J. A. (2000). Evidence for myocardial ATP compartmentation from NMR inversion transfer analysis of creatine kinase fluxes. Biophys. J. 79, 1–13 10866933 10.1016/s0006-3495(00)76269-2 PMC1300911

[B64] Joubert F. Hoerter J. A. Mazet J. L. (2001a). Discrimination of cardiac subcellular creatine kinase fluxes by NMR spectroscopy: a new method of analysis. Biophys. J. 81, 2995–3004 10.1016/S0006-3495(01)75940-1 11720970 PMC1301764

[B65] Joubert F. Vrezas I. Mateo P. Gillet B. Beloeil J. C. Soboll S. (2001b). Cardiac creatine kinase metabolite compartments revealed by NMR magnetization transfer spectroscopy and subcellular fractionation. Biochemistry 40, 2129–2137 10.1021/bi001695j 11329281

[B66] Joubert F. Mazet J. L. Mateo P. Hoerter J. A. (2002a). 31P NMR detection of subcellular creatine kinase fluxes in the perfused rat heart: contractility modifies energy transfer pathways. J. Biol. Chem. 277, 18469–18476 10.1074/jbc.M200792200 11886866

[B67] Joubert F. Hoerter J. A. Mazet J. L. (2002b). Modeling the energy transfer pathways. creatine kinase activities and heterogeneous distribution of ADP in the perfused heart. Mol. Biol. Rep. 29, 177–182 12241053 10.1023/a:1020321711771

[B68] Joubert F. Mazet J. L. Mateo P. Hoerter J. A. (2002c). Identification of subcellular energy fluxes by P NMR spectroscopy in the perfused heart: contractility induced modifications of energy transfer pathways. Mol. Biol. Rep. 29, 171–176 12241052 10.1023/a:1020369627701

[B69] Joubert F. Wilding J. R. Fortin D. Domergue-Dupont V. Novotova M. Ventura-Clapier R. (2008). Local energetic regulation of sarcoplasmic and myosin ATPase is differently impaired in rats with heart failure. J. Physiol. 586, 5181–5192 10.1113/jphysiol.2008.157677 18787038 PMC2652147

[B70] Kaasik A. Joubert F. Ventura Clapier R. Veksler V. (2004). A novel mechanism of regulation of cardiac contractility by mitochondrial functional state. FASEB J. 18, 1219–1227 10.1096/fj.04-1508com 15284222

[B71] Kaasik A. Kuum M. Joubert F. Wilding J. Ventura-Clapier R. Veksler V. (2010). Mitochondria as a source of mechanical signals in cardiomyocytes. Cardiovasc. Res. 87, 83–91 10.1093/cvr/cvq039 20124402

[B72] Kaasik A. Veksler V. Boehm E. Novotova M. Minajeva A. Ventura-Clapier R. (2001). Energetic crosstalk between organelles: architectural integration of energy production and utilization. Circ. Res. 89, 153–159 10.1161/hh1401.093440 11463722

[B73] Karbowski M. Youle R. J. (2003). Dynamics of mitochondrial morphology in healthy cells and during apoptosis. Cell Death Differ. 10, 870–880 10.1038/sj.cdd.4401260 12867994

[B74] Khalifat N. Puff N. Bonneau S. Fournier J. B. Angelova M. I. (2008). Membrane deformation under local pH gradient: mimicking mitochondrial cristae dynamics. Biophys. J. 95, 4924–4933 10.1529/biophysj.108.136077 18689447 PMC2576396

[B75] Kimberg D. V. Loeb J. N. (1972). Effects of cortisone administration on rat liver mitochondria. Support for the concept of mitochondrial fusion. J. Cell Biol. 55, 635–643 4656705 10.1083/jcb.55.3.635 PMC2108811

[B76] Koch A. Thiemann M. Grabenbauer M. Yoon Y. McNiven M. A. Schrader M. (2003). Dynamin-like protein 1 is involved in peroxisomal fission. J. Biol. Chem. 278, 8597–8605 10.1074/jbc.M211761200 12499366

[B77] Koch A. Yoon Y. Bonekamp N. A. McNiven M. A. Schrader M. (2005). A role for Fis1 in both mitochondrial and peroxisomal fission in mammalian cells. Mol. Biol. Cell 16, 5077–5086 10.1091/mbc.E05-02-0159 16107562 PMC1266408

[B78] Koshiba T. Detmer S. A. Kaiser J. T. Chen H. McCaffery J. M. Chan D. C. (2004). Structural basis of mitochondrial tethering by mitofusin complexes. Science 305, 858–862 10.1126/science.1099793 15297672

[B79] Kuznetsov A. V. Hermann M. Saks V. Hengster P. Margreiter R. (2009). The cell-type specificity of mitochondrial dynamics. Int. J. Biochem. Cell Biol. 41, 1928–1939 10.1016/j.biocel.2009.03.007 19703655

[B80] Lee Y. Lee H. Y. Hanna R. A. Gustafsson A. B. (2011). Mitochondrial autophagy by Bnip3 involves Drp1-mediated mitochondrial fission and recruitment of Parkin in cardiac myocytes. Am. J. Physiol. Heart Circ. Physiol. 301, H1924–H1931 10.1152/ajpheart.00368.2011 21890690 PMC3213962

[B81] Lee Y. J. Jeong S. Y. Karbowski M. Smith C. L. Youle R. J. (2004). Roles of the mammalian mitochondrial fission and fusion mediators Fis1, Drp1, and Opa1 in apoptosis. Mol. Biol. Cell 15, 5001–5011 10.1091/mbc.E04-04-0294 15356267 PMC524759

[B82] Legros F. Lombes A. Frachon P. Rojo M. (2002). Mitochondrial fusion in human cells is efficient, requires the inner membrane potential, and is mediated by mitofusins. Mol. Biol. Cell 13, 4343–4354 10.1091/mbc.E02-06-0330 12475957 PMC138638

[B83] Leu M. Ehler E. Perriard J. C. (2001). Characterisation of postnatal growth of the murine heart. Anat. Embryol. (Berl.) 204, 217–224 11681801 10.1007/s004290100206

[B84] Liesa M. Palacin M. Zorzano A. (2009). Mitochondrial dynamics in mammalian health and disease. Physiol. Rev. 89, 799–845 10.1152/physrev.00030.2008 19584314

[B85] Mannella C. A. (2006). Structure and dynamics of the mitochondrial inner membrane cristae. Biochim. Biophys. Acta 1763, 542–548 10.1016/j.bbamcr.2006.04.006 16730811

[B86] Mannella C. A. (2008). Structural diversity of mitochondria: functional implications. Ann. N.Y. Acad. Sci. 1147, 171–179 10.1196/annals.1427.020 19076440 PMC2605638

[B87] Messerschmitt M. Jakobs S. Vogel F. Fritz S. Dimmer K. S. Neupert W. (2003). The inner membrane protein Mdm33 controls mitochondrial morphology in yeast. J. Cell Biol. 160, 553–564 10.1083/jcb.200211113 12591915 PMC2173741

[B88] Narula J. Pandey P. Arbustini E. Haider N. Narula N. Kolodgie F. D. (1999). Apoptosis in heart failure: release of cytochrome c from mitochondria and activation of caspase-3 in human cardiomyopathy. Proc. Natl. Acad. Sci. U.S.A. 96, 8144–8149 10.1073/pnas.96.14.8144 10393962 PMC22202

[B89] Ngoh G. A. Papanicolaou K. N. Walsh K. (2012). Loss of mitofusin 2 promotes endoplasmic reticulum stress. J. Biol. Chem. 287, 20321–20332 10.1074/jbc.M112.359174 22511781 PMC3370214

[B90] Nogueira V. Devin A. Walter L. Rigoulet M. Leverve X. Fontaine E. (2005). Effects of decreasing mitochondrial volume on the regulation of the permeability transition pore. J. Bioenerg. Biomembr. 37, 25–33 10.1007/s10863-005-4120-3 15906146

[B91] Olichon A. Baricault L. Gas N. Guillou E. Valette A. Belenguer P. (2003). Loss of OPA1 perturbates the mitochondrial inner membrane structure and integrity, leading to cytochrome c release and apoptosis. J. Biol. Chem. 278, 7743–7746 10.1074/jbc.C200677200 12509422

[B92] Olichon A. Guillou E. Delettre C. Landes T. Arnaune-Pelloquin L. Emorine L. J. (2006). Mitochondrial dynamics and disease, OPA1. Biochim. Biophys. Acta. 1763, 500–509 10.1016/j.bbamcr.2006.04.003 16737747

[B93] Olichon A. Landes T. Arnaune-Pelloquin L. Emorine L. J. Mils V. Guichet A. (2007). Effects of OPA1 mutations on mitochondrial morphology and apoptosis: relevance to ADOA pathogenesis. J. Cell Physiol. 211, 423–430 10.1002/jcp.20950 17167772

[B94] Olivetti G. Abbi R. Quaini F. Kajstura J. Cheng W. Nitahara J. A. (1997). Apoptosis in the failing human heart. N. Engl. J. Med. 336, 1131–1141 10.1056/NEJM199704173361603 9099657

[B95] Ong S. B. Hall A. R. Hausenloy D. J. (2012). Mitochondrial dynamics in cardiovascular health and disease. Antioxid. Redox. Signal. [Epub ahead of print]. 10.1089/ars.2012.4777 22793879 PMC3699895

[B96] Ong S. B. Subrayan S. Lim S. Y. Yellon D. M. Davidson S. M. Hausenloy D. J. (2010). Inhibiting mitochondrial fission protects the heart against ischemia/reperfusion injury. Circulation 121, 2012–2022 10.1161/CIRCULATIONAHA.109.906610 20421521

[B97] Otera H. Wang C. Cleland M. M. Setoguchi K. Yokota S. Youle R. J. (2010). Mff is an essential factor for mitochondrial recruitment of Drp1 during mitochondrial fission in mammalian cells. J. Cell Biol. 191, 1141–1158 10.1083/jcb.201007152 21149567 PMC3002033

[B98] Palmer C. S. Osellame L. D. Laine D. Koutsopoulos O. S. Frazier A. E. Ryan M. T. (2011). MiD49 and MiD51, new components of the mitochondrial fission machinery. EMBO Rep. 12, 565–573 10.1038/embor.2011.54 21508961 PMC3128275

[B99] Papanicolaou K. N. Khairallah R. J. Ngoh G. A. Chikando A. Luptak I. O'Shea K. M. (2011). Mitofusin-2 maintains mitochondrial structure and contributes to stress-induced permeability transition in cardiac myocytes. Mol. Cell Biol. 31, 1309–1328 10.1128/MCB.00911-10 21245373 PMC3067905

[B100] Papanicolaou K. N. Ngoh G. A. Dabkowski E. R. O'Connell K. A. Ribeiro R. F. Jr. Stanley W. C. (2012). Cardiomyocyte deletion of mitofusin-1 leads to mitochondrial fragmentation and improves tolerance to ROS-induced mitochondrial dysfunction and cell death. Am. J. Physiol. Heart Circ. Physiol. 302, H167–H179 10.1152/ajpheart.00833.2011 22037195 PMC3334239

[B101] Parra V. Eisner V. Chiong M. Criollo A. Moraga F. Garcia A. (2008). Changes in mitochondrial dynamics during ceramide-induced cardiomyocyte early apoptosis. Cardiovasc. Res. 77, 387–397 10.1093/cvr/cvm029 18006463

[B102] Parra V. Verdejo H. del Campo A. Pennanen C. Kuzmicic J. Iglewski M. (2011). The complex interplay between mitochondrial dynamics and cardiac metabolism. J. Bioenerg. Biomembr. 43, 47–51 10.1007/s10863-011-9332-0 21258852 PMC3286637

[B103] Pich S. Bach D. Briones P. Liesa M. Camps M. Testar X. (2005). The Charcot-Marie-Tooth type 2A gene product, Mfn2, up-regulates fuel oxidation through expression of OXPHOS system. Hum. Mol. Genet. 14, 1405–1415 10.1093/hmg/ddi149 15829499

[B104] Piquereau J. Caffin F. Novotova M. Prola A. Garnier A. Mateo P. (2012). Down-regulation of OPA1 alters mouse mitochondrial morphology, PTP function, and cardiac adaptation to pressure overload. Cardiovasc. Res. 94, 408–417 10.1093/cvr/cvs117 22406748 PMC3863708

[B105] Piquereau J. Novotova M. Fortin D. Garnier A. Ventura-Clapier R. Veksler V. (2010). Postnatal development of mouse heart: formation of energetic microdomains. J. Physiol. 588, 2443–2454 10.1113/jphysiol.2010.189670 20478976 PMC2915519

[B106] Piquereau J. Novotova M. Garnier A. Joubert F. Veksler V. Ventura-Clapier R. (2013). Cardiac metabolic adaptation during postnatal development, in Cardiac Adaptations, Advances in Biochemistry in Health and Disease, Vol. 4, eds Ostadal B. Dhalla N. S. (New York, NY: Springer), 79–98

[B107] Rappaport L. Oliviero P. Samuel J. L. (1998). Cytoskeleton and mitochondrial morphology and function. Mol. Cell Biochem. 184, 101–105 9746315

[B108] Rossignol R. Gilkerson R. Aggeler R. Yamagata K. Remington S. J. Capaldi R. A. (2004). Energy substrate modulates mitochondrial structure and oxidative capacity in cancer cells. Cancer Res. 64, 985–993 10.1158/0008-5472.CAN-03-1101 14871829

[B109] Sabbah H. N. Sharov V. Riddle J. M. Kono T. Lesch M. Goldstein S. (1992). Mitochondrial abnormalities in myocardium of dogs with chronic heart failure. J. Mol. Cell Cardiol. 24, 1333–1347 10.1016/0022-2828(92)93098-5 1479624

[B110] Saks V. A. Kaambre T. Sikk P. Eimre M. Orlova E. Paju K. (2001). Intracellular energetic units in red muscle cells. Biochem. J. 356, 643–657 11368796 10.1042/0264-6021:3560643 PMC1221880

[B111] Saks V. A. Kongas O. Vendelin M. Kay L. (2000). Role of the creatine/phosphocreatine system in the regulation of mitochondrial respiration. Acta Physiol. Scand. 168, 635–641 10.1046/j.1365-201x.2000.00715.x 10759600

[B112] Santel A. Frank S. Gaume B. Herrler M. Youle R. J. Fuller M. T. (2003). Mitofusin-1 protein is a generally expressed mediator of mitochondrial fusion in mammalian cells. J. Cell Sci. 116, 2763–2774 10.1242/jcs.00479 12759376

[B113] Santel A. Fuller M. T. (2001). Control of mitochondrial morphology by a human mitofusin. J. Cell Sci. 114, 867–874 11181170 10.1242/jcs.114.5.867

[B114] Satoh M. Hamamoto T. Seo N. Kagawa Y. Endo H. (2003). Differential sublocalization of the dynamin-related protein OPA1 isoforms in mitochondria. Biochem. Biophys. Res. Commun. 300, 482–493 10.1016/S0006-291X(02)02874-7 12504110

[B115] Schaper J. Froede R. Hein S. Buck A. Hashizume H. Speiser B. (1991). Impairment of the myocardial ultrastructure and changes of the cytoskeleton in dilated cardiomyopathy. Circulation 83, 504–514 10.1161/01.CIR.83.2.504 1991369

[B114a] Shahrestani P. Leung H. T. Le P. K. Pak W. L. Tse S. Ocorr K. (2009). Heterozygous mutation of Drosophila Opa1 causes the development of multiple organ abnormalities in an age-dependent and organ-specific manner. PLos One 4:e6867 10.1371/journal.pone.0006867 19718456 PMC2730818

[B116] Shen T. Zheng M. Cao C. Chen C. Tang J. Zhang W. (2007). Mitofusin-2 is a major determinant of oxidative stress-mediated heart muscle cell apoptosis. J. Biol. Chem. 282, 23354–23361 10.1074/jbc.M702657200 17562700

[B117] Skulachev V. P. (2001). Mitochondrial filaments and clusters as intracellular power-transmitting cables. Trends Biochem. Sci. 26, 23–29 10.1016/S0968-0004(00)01735-7 11165513

[B118] Soubannier V. McBride H. M. (2009). Positioning mitochondrial plasticity within cellular signaling cascades. Biochim. Biophys. Acta 1793, 154–170 10.1016/j.bbamcr.2008.07.008 18694785

[B119] Speer O. Back N. Buerklen T. Brdiczka D. Koretsky A. Wallimann T. (2005). Octameric mitochondrial creatine kinase induces and stabilizes contact sites between the inner and outer membrane. Biochem. J. 385, 445–450 10.1042/BJ20040386 15294016 PMC1134715

[B120] Stojanovski D. Koutsopoulos O. S. Okamoto K. Ryan M. T. (2004). Levels of human Fis1 at the mitochondrial outer membrane regulate mitochondrial morphology. J. Cell Sci. 117, 1201–1210 10.1242/jcs.01058 14996942

[B121] Strauss M. Hofhaus G. Schroder R. R. Kuhlbrandt W. (2008). Dimer ribbons of ATP synthase shape the inner mitochondrial membrane. EMBO J. 27, 1154–1160 10.1038/emboj.2008.35 18323778 PMC2323265

[B122] Tepp K. Shevchuk I. Chekulayev V. Timohhina N. Kuznetsov A. V. Guzun R. (2011). High efficiency of energy flux controls within mitochondrial interactosome in cardiac intracellular energetic units. Biochim. Biophys. Acta 1807, 1549–1561 10.1016/j.bbabio.2011.08.005 21872567

[B123] Tieu Q. Okreglak V. Naylor K. Nunnari J. (2002). The WD repeat protein, Mdv1p, functions as a molecular adaptor by interacting with Dnm1p and Fis1p during mitochondrial fission. J. Cell Biol. 158, 445–452 10.1083/jcb.200205031 12163467 PMC2173813

[B124] Tondera D. Czauderna F. Paulick K. Schwarzer R. Kaufmann J. Santel A. (2005). The mitochondrial protein MTP18 contributes to mitochondrial fission in mammalian cells. J. Cell Sci. 118, 3049–3059 10.1242/jcs.02415 15985469

[B125] Twig G. Elorza A. Molina A. J. Mohamed H. Wikstrom J. D. Walzer G. (2008a). Fission and selective fusion govern mitochondrial segregation and elimination by autophagy. EMBO J. 27, 433–446 10.1038/sj.emboj.7601963 18200046 PMC2234339

[B126] Twig G. Hyde B. Shirihai O. S. (2008b). Mitochondrial fusion, fission and autophagy as a quality control axis: the bioenergetic view. Biochim. Biophys. Acta 1777, 1092–1097 10.1016/j.bbabio.2008.05.001 18519024 PMC3809017

[B127] Vafai S. B. Mootha V. K. (2012). Mitochondrial disorders as windows into an ancient organelle. Nature 491, 374–383 10.1038/nature11707 23151580

[B128] Varadi A. Johnson-Cadwell L. I. Cirulli V. Yoon Y. Allan V. J. Rutter G. A. (2004). Cytoplasmic dynein regulates the subcellular distribution of mitochondria by controlling the recruitment of the fission factor dynamin-related protein-1. J. Cell Sci. 117, 4389–4400 10.1242/jcs.01299 15304525

[B129] Vendelin M. Beraud N. Guerrero K. Andrienko T. Kuznetsov A. V. Olivares J. (2005). Mitochondrial regular arrangement in muscle cells: a “crystal-like” pattern. Am. J. Physiol. Cell Physiol. 288, C757–C767 10.1152/ajpcell.00281.2004 15496480

[B130] Ventura-Clapier R. Garnier A. Veksler V. Joubert F. (2011). Bioenergetics of the failing heart. Biochim. Biophys. Acta 1813, 1360–1372 10.1016/j.bbamcr.2010.09.006 20869993

[B131] Wakabayashi T. (2002). Megamitochondria formation – physiology and pathology. J. Cell Mol. Med. 6, 497–538 10.1111/j.1582-4934.2002.tb00452.x 12611638 PMC6741312

[B132] Wakabayashi J. Zhang Z. Wakabayashi N. Tamura Y. Fukaya M. Kensler T. W. (2009). The dynamin-related GTPase Drp1 is required for embryonic and brain development in mice. J. Cell Biol. 186, 805–816 10.1083/jcb.200903065 19752021 PMC2753156

[B133] Wakabayashi T. Asano M. Kurono C. (1975). Mechanism of the formation of megamitochondria induced by copper-chelating agents. I. On the formation process of megamitochondria in cuprizone-treated mouse liver. Acta Pathol. Jpn. 25, 15–37 1136774 10.1111/j.1440-1827.1975.tb00147.x

[B134] Wakabayashi T. Green D. E. (1977). Membrane fusion in mitochondria. I. Ultrastructural basis for fusion. J. Electron Microsc. (Tokyo) 26, 305–320 604413

[B135] Wallimann T. Wyss M. Brdiczka D. Nicolay K. Eppenberger H. M. (1992). Intracellular compartmentation, structure and function of creatine kinase isoenzymes in tissues with high and fluctuating energy demands: the ‘phosphocreatine circuit’ for cellular energy homeostasis. Biochem. J. 281(Pt 1), 21–40 1731757 10.1042/bj2810021 PMC1130636

[B136] Wasiak S. Zunino R. McBride H. M. (2007). Bax/Bak promote sumoylation of DRP1 and its stable association with mitochondria during apoptotic cell death. J. Cell Biol. 177, 439–450 10.1083/jcb.200610042 17470634 PMC2064824

[B137] Wasilewski M. Scorrano L. (2009). The changing shape of mitochondrial apoptosis. Trends Endocrinol. Metab. 20, 287–294 10.1016/j.tem.2009.03.007 19647447

[B138] Wilding J. R. Joubert F. de Araujo C. Fortin D. Novotova M. Veksler V. (2006). Altered energy transfer from mitochondria to sarcoplasmic reticulum after cytoarchitectural perturbations in mice hearts. J. Physiol. 575, 191–200 10.1113/jphysiol.2006.114116 16740607 PMC1819422

[B139] Yoon Y. Krueger E. W. Oswald B. J. McNiven M. A. (2003). The mitochondrial protein hFis1 regulates mitochondrial fission in mammalian cells through an interaction with the dynamin-like protein DLP1. Mol. Cell Biol. 23, 5409–5420 10.1128/MCB.23.15.5409-5420.2003 12861026 PMC165727

[B140] Yoon Y. Pitts K. R. McNiven M. A. (2001). Mammalian dynamin-like protein DLP1 tubulates membranes. Mol. Biol. Cell 12, 2894–2905 11553726 10.1091/mbc.12.9.2894 PMC59722

[B141] Zhao T. Huang X. Han L. Wang X. Cheng H. Zhao Y. (2012). Central role of mitofusin 2 in autophagosome-lysosome fusion in cardiomyocytes. J. Biol. Chem. 287, 23615–23625 10.1074/jbc.M112.379164 22619176 PMC3390636

[B142] Ziviani E. Tao R. N. Whitworth A. J. (2010). Drosophila parkin requires PINK1 for mitochondrial translocation and ubiquitinates mitofusin. Proc. Natl. Acad. Sci. U.S.A. 107, 5018–5023 10.1073/pnas.0913485107 20194754 PMC2841909

[B143] Ziviani E. Whitworth A. J. (2010). How could Parkin-mediated ubiquitination of mitofusin promote mitophagy? Autophagy 6, 660–662 10.4161/auto.6.5.12242 20484985 PMC4196639

[B144] Zuchner S. Mersiyanova I. V. Muglia M. Bissar-Tadmouri N. Rochelle J. Dadali E. L. (2004). Mutations in the mitochondrial GTPase mitofusin 2 cause Charcot-Marie-Tooth neuropathy type 2A. Nat. Genet. 36, 449–451 10.1038/ng1341 15064763

